# Changes in, and factors associated with family functioning: results of four cross-sectional household surveys from 2011 to 2017 in Hong Kong

**DOI:** 10.1186/s12889-024-17643-6

**Published:** 2024-01-11

**Authors:** Camilla Kin Ming Lo, Qiqi Chen, Mengtong Chen, Ko Ling Chan, Patrick Ip

**Affiliations:** 1 Department of Applied Social Sciences, The Hong Kong Polytechnic University, Hung Hom, Hong Kong, China; 2 Department of Social Work, The Chinese University of Hong Kong, Shatin, Hong Kong China; 3 Department of Paediatrics & Adolescent Medicine, The University of Hong Kong, Hong Kong, China

**Keywords:** Family functioning, Family structures, Household survey, Cross-sectional

## Abstract

Previous research has well-documented that family functioning is an important predictor of individuals’ physical and mental health. However, relatively little research has explored family functioning at the family and population levels, such as changes in family functioning across years and whether predictors of family functioning differ across different family structures. Understanding of the changes in family functioning across years and factors promoting family functioning will inform the development of preventive measures to enhance family health and resilience. Objectives of the study were: (1) to examine the changes in family functioning across a 6-year study period and (2) to study protective factors associated with family functioning and the extent to which the factors are the same or different across different family structures. Method: The study involved secondary data analysis of the biannual Family Survey carried out by the Family Council, an advisory body to the Hong Kong government. A series of ANOVA and regression analyses were conducted using data of four population-based cross-sectional household surveys conducted in Hong Kong in 2011, 2013, 2015, and 2017. Results: There were some fluctuations in overall family functioning scores across the study period, but no significant difference was found between the scores in 2011 and 2017. Different predictors of family functioning were found for different family types, and frequent family communication was a common protective factor for most family types, including never married, married/cohabiting with children, and married/cohabiting with no children. Conclusion: The study is among the first to examine changes in family functioning at the population level. Monitoring and addressing family functioning may help tackle various social problems and future public health crises. Interventions to promote family functioning should address both common and different protective factors of different family types.

**Supplementary Information:**

The online version contains supplementary material available at 10.1186/s12889-024-17643-6.

## Highlights

A secondary analysis of population-based cross-sectional household surveys revealed no significant changes in family functioning in Hong Kong between 2011 and 2017, despite some fluctuations across the years.

Family communication is a common factor associated with higher family functioning in most family types.

Different protective factors are found for specific family types, suggesting interventions should take family types into account to effectively strengthen family functioning.

## Introduction

From a family systems perspective [1], family members’ interactions shape the behaviors of individual members and individual members contribute to the overall well-being of the family. Such interconnectedness means that any impairment in the functioning at the family level may lead to changes in the functioning of individual members. Family functioning, which is the extent to which family members are able to resolve problems and communicate effectively and ensure proper role allocation, emotional response and involvement within the family [2], has been widely studied in understanding individuals’ physical and mental health [3, 4] and it is considered one of the key contributors to life satisfaction [5]. In the study of children and adolescents, there has been mounting evidence supporting the relationship between family functioning and various outcomes such as children and youth’s substance use [6], internalizing and externalizing behaviors [7, 8], depression [9], and risk of maltreatment [10]. Given that family functioning is so closely related to individuals’ physical and mental health, scholars have argued that family factors should be considered as a key social determinants of health [11]. In a broader context, routine data collection and monitoring of family functioning at the population level has long been advocated for to inform decision making in addressing social and public health issues [12], but relevant research has been very little. The most relevant work is the Australia’s National Framework for Protecting Australia’s Children (NFPAC), which includes measures that report on children’s safety and well-being at the population level [13]. Within this framework, family functioning is one of the outcome indicators of whether children are living in safe and supportive families and communities, and routine data on family functioning has been collected since 2010 to inform longer-term policy planning [13]. Apart from the experience in Australia, no other studies have examined the trends in family functioning in the general population across time.

During the global COVID-19 pandemic, numerous studies have found that social distancing measures had profound impacts on family life and family functioning [14, 15]. Meanwhile, higher levels of family functioning buffered against negative impacts of the pandemic, such as children’s quality of life [16]. One of the important lessons learned from the pandemic is that we should establish preventive measures to promote individual and family resilience before occurrence of a crisis [17]. Hence, research to identify factors that predict family functioning will provide useful information for the development of such preventive measures. According to the Stress Process Model [18, 19], predictors of family functioning can be categorized into contextual variables, primary stressors, secondary stressors, and resources. Contextual variables include demographic characteristics, primary stressors are sources of stress, and secondary stressors are responses to the primary stressors and are usually interpersonal in nature. The Stress Process Model concerns both the stressors and the availability of coping resources associated with family functioning. In the family functioning literature, research work has primarily focused on the stressors (risk factors) that impair the functioning of families, including work-family conflict [20, 21], chronic illnesses of family members [22, 23], and interparental conflict [24]. However, relatively little research work has evaluated the resource (protective) factors that promote family functioning. Among the relevant studies, social support, including perceived levels of social support and actual utilization of support, are found to be an important predictor of family functioning [25, 26]. Apart from social support, spending more time with family members such as in form of family meals and family-based activities are also found to be associated with increased family connectedness [27–29]. Frequency of communication is another potential protective factor for family functioning. Recent studies looked into the benefits of family communication via new technologies, showing that more daily messages exchanged between family members via instant messengers is associated increased family functioning [30].

To add an additional layer to the study of predictors of family functioning, there is a need to examine how these predictors differ by family structures. Research has paid increasing attention to family structure in the study of family functioning [31]. Family structure is a term used to describe the type of household in which members are related to each other by marital/partnership status or bloodline [32]. Viewing from a social capital perspective, households of different family structures may have different levels of intra and extra-familial social networks, which may influence family process and subsequently family functioning [31]. Past studies suggested a relationship between different types of family structure and family functioning, albeit inconclusive [3, 33, 34]. A more recent study conducted during the pandemic found lower levels of family functioning in families without children, compared with families with children [34]. As the functioning of a family may be related to its structure, it is important to explore the similarities and differences of the predictors of family functioning across different family structures. Such understanding would encourage the development of tailored interventions to promote family functioning for different types of family, which in turn better benefit individuals’ well-being and development.

Regarding the research context, family and family functioning may be more influential to populations with a strong emphasis on collectivist values and the importance of interdependence among family members. Taking Hong Kong as an example, although it is the most westernized city in China, traditional cultural values about the importance of family, family hierarchy and harmony underlie many aspects of family lives [35]. Exploring the family functioning construct in families in Hong Kong will provide useful reference to inform further study and development of preventive programs to improve family health and resilience in other collectivist cultures with similar socioeconomic backgrounds. Using data from four representative household surveys in Hong Kong, the objectives of this study were (1) to explore the changes in family functioning in Hong Kong from 2011 to 2017 and (2) to examine the resource (protective) factors associated with family functioning and the extent to which these factors are the same or different across various family structures.

## Method

### Sample

Using data from the Family Survey, a territory-wide household questionnaire, a secondary analysis was conducted to investigate changes in family functioning across years and the various predictors of family functioning among general families in Hong Kong. The Family Survey adopted cross-sectional study design and was carried out by the Family Council, an advisory body to the Hong Kong government, on a biannual basis in 2011, 2013, 2015, and 2017 [36]. These surveys provided updated and evidence-based data on changes and developments among Hong Kong families in regard to parenthood, family functioning, satisfaction with family life, work-family balance, social support networks, and awareness of and participation in family-related programs. The biannual Family Survey targeted all persons aged 15 or above residing in Hong Kong. A two-stage stratified random sampling design was adopted to select participants. For the first stage, a list of living quarters obtained from the Census & Statistics Department was randomly selected by geographical area and type of quarter. For the second stage, a household member aged 15 years or above in each household was randomly selected for completing the cross-sectional survey using the last birthday method. The data of the current study included 8,932 representative households of the biannual Family Surveys. Specifically, 2000, 2000, 2000, and 2932 participants from different households were recruited in 2011, 2013, 2015, and 2017 respectively.

### Measures

#### Demographic characteristics

Several demographic variables, including age, education level, and family structure were reported. Education attainment was coded as primary education or lower, secondary education, and postsecondary education or above. Family structure was coded as (1) never married, (2) married/cohabiting with no children, (3) married/cohabiting with children, (4) divorced/separated, and (5) widowed.

#### Family functioning (outcome variable)

Family functioning was assessed as the outcome variable using the Chinese Family Assessment Instrument (CFAI) [37]. The CFAI is a 33-item, validated measurement consisting of five subscales: mutuality (twelve items) (α > 0.07), communication (nine items) (α > 0.07), conflict and harmony (six items) (α > 0.07), parental concern (three items) (α > 0.07), and parental control (three items) (α > 0.07). Sample questions include: “family members support each other” (mutuality); “family members enjoy getting together” (communication); “not many quarrels among family members” (conflict and harmony); “parents take care of their children” (parental concern); and “parents’ control is too harsh” (parental control). Respondents were asked to indicate their level of agreement with each item on a five-point Likert scale, ranging from 1 (*does not fit our family*) to 5 (*very much fits our family*). For the mutuality, communication, and parental concern subscales, a higher score represented higher levels of mutual concern among family members and better family relationship and communication. For the conflict and harmony and parental control dimensions, a higher score indicated lower levels of family conflict and parental control over children. In addition to the five subscales, an overall family functioning score was computed by taking the mean score of all 33 items; a higher score indicated better family functioning.

#### Family gathering activities (predictors)

##### Family meal frequency (3 items)

Participants were asked to rate the frequency of family meals with their mother, father, and spouse/partner, respectively, on a four-point Likert scale ranging from 1 (*almost never*) to 4 (*always*). The scores were then averaged to give an overall value. A value higher than 2 was coded as a high frequency of family meals while lower than 2 was coded as a low frequency.

##### Family gathering frequency (3 items)

Participants were asked to rate the frequency of family gatherings with their mother, father, and spouse/partner, respectively, on a four-point Likert scale, ranging from 1 (*almost never*) to 4 (*always*). The scores were then averaged to give an overall value. A value higher than 2 was coded as a high frequency of family gatherings while lower than 2 was coded as a low frequency.

##### Time spent with parents (1 item)

Participants were asked to report the amount of time they spent with their parent(s) talking about something important to them during the week. The question item was coded as follows: 1 (*never*), 2 (*less than 5 min*), 3 (*5 to 15 min*), 4 (*16 to 30 min*), 5 (*31 to 60 min*), 6 (*1 h to less than 2 h*), 7 (*2 h to less than 4 h*), and 8 (*4 h or above*). A value of 1 was coded as did not spend time with parents, and values 2 to 8 were coded as had spent time with parents.

##### Time spent with parents or spouse/partner (1 item)

Participants were asked to report the amount of time they spent with their parent(s) or spouse/partner talking about something important to them during the week. The question item was code as follows: 1 (*never*), 2 (*less than 5 min*), 3 (*5 to 15 min*), 4 (*16 to 30 min*), 5 (*31 to 60 min*), 6 (*1 h to less than 2 h*), 7 (*2 h to less than 4 h*), and 8 (*4 h or above*). A value of 1 was coded as did not spend time with parents or spouse/partner, and values of 2 to 8 were coded as had spent time with parents or spouse/partner.

#### Formal and informal social support (predictors)

##### Informal social support from family members (1 item)

Participants were asked whether they turned to their family members if they experienced emotional and financial difficulties. Participants reporting “yes” to the question were regarded as having received such support.

##### Informal social support from friends, neighbors, and coworkers (1 item)

Participants were asked whether they turned to friends, neighbors, or coworkers if they experienced emotional and financial difficulties. Participants reporting “yes” to the question were regarded as having received informal social support.

##### Formal social support (1 item)

Participants were asked whether they turned to formal services, including government departments, nongovernmental organizations, and religious groups, if they experienced emotional and financial difficulties. Participants reporting “yes” to the question were regarded as having received formal social support.

#### Family communication (predictors)

##### Frequency of communication with family members and between generations via technology

Participants were asked to rate the frequency of using technology to communicate with family members and between generations on a four-point Likert scale ranging from 1 (*almost never*) to 4 (*always*). The scores were then averaged to give an overall value. A higher score indicated more frequent family and intergenerational communication via technology.

##### Frequency of communication with family members and between generations

Participants were asked to rate how often they communicated with family members and between generations on a four-point Likert scale ranging from 1 (*almost never*) to 4 (*always*). The scores were then averaged to give an overall value. A higher score indicated more frequent communication.

### Ethics approval

The study involved secondary data analysis, and ethics approval was given by the Home Affairs Bureau of the Government of the Hong Kong Special Administrative Region. Informed consent was obtained from all participants involved in the study.

### Data analysis

Descriptive analyses were first conducted to summarize and compare the participants’ demographic characteristics for different years, including age, gender, education level, and family structure. Then, a series of ANOVA analyses were performed to examine differences in overall family functioning and the subscales of family functioning across years. To provide more in-depth information about between-year mean differences in family functioning, a series of t-test analyses were conducted. Additionally, a series of ANOVA analyses on the overall and subscales of family functioning were performed for different types of family structure and the results are presented in Additional File 1. Finally, the data of the four cross-sectional surveys were combined and logistic regression analyses were conducted to examine the associations between the predictors and family functioning for different family structures. Family functioning was coded dichotomously: scores of 4 (*fits our family*) and 5 (*very much fits our family*) were coded as high functioning, and scores of 1 to 3 were coded as low family functioning. An odds ratio (OR) higher than 1 indicated higher odds of high functioning. Confounding variables, including age, gender, and education level, were adjusted in the regression analyses. For the regression analyses, data of all four years were combined. As there was a small amount of missing data (approximately 1%), listwise deletion was used when conducting the data analyses. All of the statistical analyses were completed using the Statistical Package for Social Sciences (SPSS, version 25.0). The significance level was set at 0.05.

## Results

### Demographic characteristics of the participants

Table 1 shows the demographic characteritsics of the respondents by year. There were significant differences in some of these characteristics. The respondents’ mean age were 49.0 (SD = 19) in 2011, 51.0 (SD = 19) in 2013, 51.9 (SD = 18.2) in 2015, and 51.4 (19.1) in 2017, with a significant age difference. A larger proportion of respondents obtained a postsecondary education or above in 2017. In terms of family types, there were more respondents reporting never being married (28.1%) in 2017 compared with the other study years. There was no significant difference in terms of gender across the years.

**Table 1:** Demographic characteristics of the participants

	2011 (*n* = 2000)	2013 (*n* = 2000)	2015 (*n* = 2000)	2017 (*n* = 2932)	Chi-square/F-test	*p* value
**Age (** ***M, SD*** **)**	49.0 (19.0)	51.0 (19.0)	51.9 (18.2)	51.4 (19.1)	9.516	< 0.001
**Gender**						
Male	926 (46.3%)	902 (45.1%)	914 (45.7%)	1278 (43.6%)	4.131	0.248
Female	1074 (53.7%)	1098 (54.9%)	1086 (54.3%)	1654 (56.4%)		
**Education level**						
Primary education or lower	632 (31.6%)	676 (33.9%)	648 (32.6%)	850 (29.0%)	37.882	< 0.001
Secondary education	1058 (53.0%)	1014 (50.9%)	1057 (53.2%)	1505 (51.4%)		
Postsecondary education or above	307 (15.4%)	303 (15.2%)	283 (14.2%)	572 (19.5%)		
**Family type (n, n%)**						
Never married	547 (27.4%)	482 (24.1%)	518 (25.9%)	821 (28.1%)	35.613	< 0.001
Married/cohabiting with no children	153 (7.7%)	118 (5.9%)	122 (6.1%)	184 (6.3%)		
Married/cohabiting with children	952 (47.7%)	1016 (50.8%)	994 (49.7%)	1385 (47.4%)		
Divorced/separated	214 (10.7%)	256 (12.8%)	214 (10.7%)	196 (6.7%)		
Widowed	129 (6.5%)	127 (6.4%)	152 (7.6%)	334 (11.4%)		

### Changes in family functioning across years

**Fig. 1:**
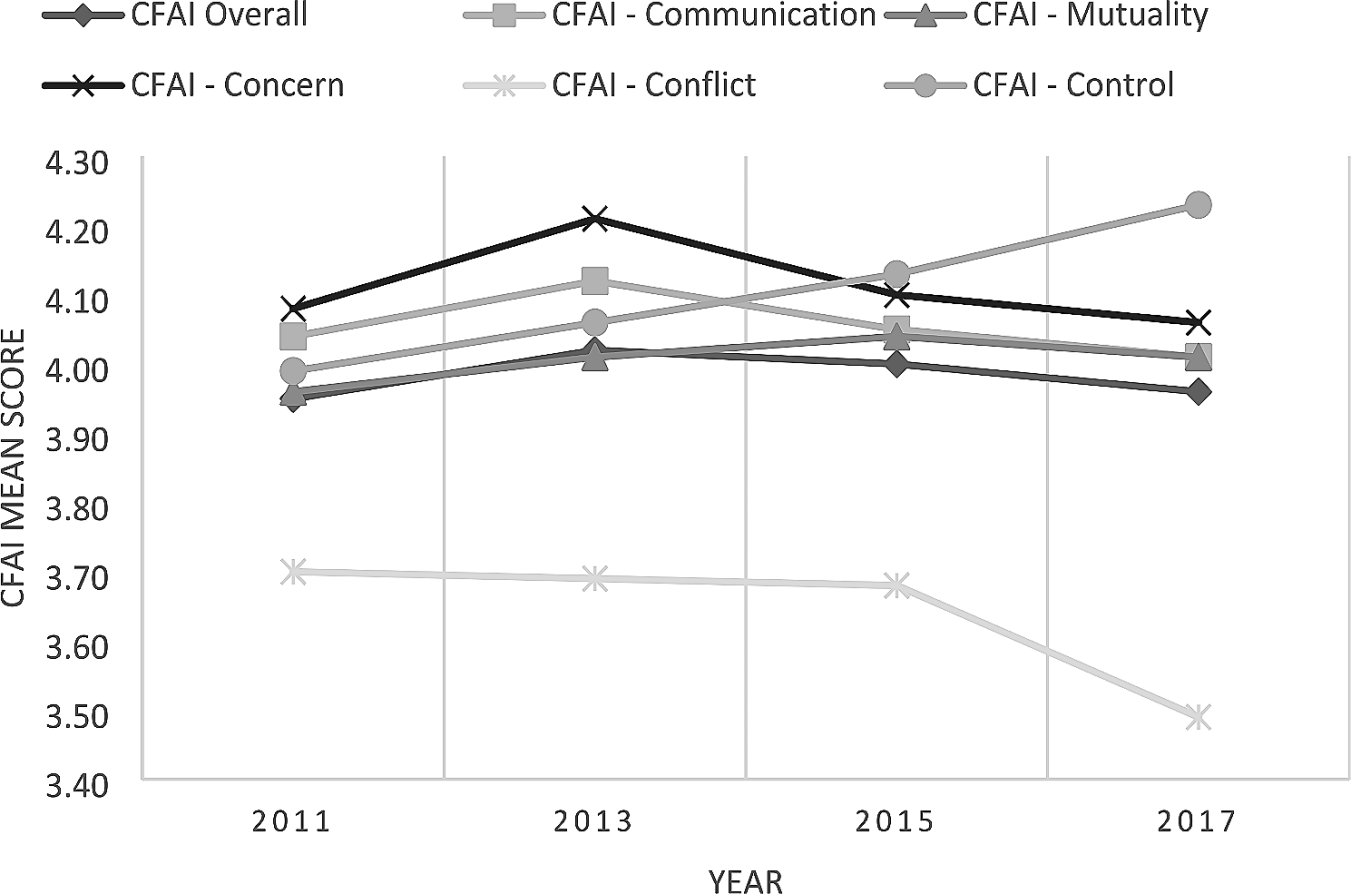
Mean scores of family functioning (CFAI) from 2011 to 2017

Figure 1; Table 2 present the changes in the mean scores of overall family functioning, measured by the CFAI and the CFAI subscales, across years. The results of the series of ANOVA and post-hoc test analyses as shown in Table 3 indicated that overall family functioning increased significantly from 2011 to 2013, then declined from 2015 to 2017. However, there was no significant difference in the overall family functioning scores between 2011 and 2017. The mean scores of the communication and concern subscales also increased significantly from 2011 to 2013, then decreased from 2013 to 2015. Similarly, the mean scores of the two subscales did not differ between 2011 and 2017. For the mutuality subscale, the mean scores did not changes between years in the short-term, but there was a siginifcant decrease in the longer term (i.e., between 2011 and 2017). The mean scores of family conflict remained stable from 2011 to 2015, followed by a significant drop in the mean score (i.e., an increase in family conflict) in 2017. There was a steady increase in the mean scores of the control subscale across the years, indicating a decreasing trend of parental control.

**Table 2:** Mean scores of family functioning (CAFI) from 2011 to 2017

	2011		2013		2015		2017		F-test	*p*	
	M	SD	M	SD	M	SD	M	SD			
CFAI Overall	3.95	0.53	4.02	0.52	4.00	0.51	3.96	0.45	8.370	< 0.001	
CFAI - Communication	4.04	0.62	4.12	0.66	4.05	0.64	4.01	0.62	12.782	< 0.001	
CFAI - Mutuality	3.96	0.62	4.01	0.61	4.04	0.58	4.01	0.52	6.929	< 0.001	
CFAI - Concern	4.08	0.69	4.21	0.67	4.10	0.65	4.06	0.57	23.068	< 0.001	
CFAI - Conflict	3.70	0.71	3.69	0.74	3.68	0.70	3.49	0.71	51.205	< 0.001	
CFAI - Control	3.99	0.73	4.06	0.72	4.13	0.77	4.23	0.65	48.036	< 0.001	

**Table 3:** Comparison of family functioning (CAFI) across years (2011–2017)

	2011 vs. 2013		2011 vs. 2015		2011 vs. 2017		2013 vs. 2015		2013 vs. 2017		2015 vs. 2017	
	*Mean difference*	*p*	*Mean difference*	*p*	*Mean difference*	*p*	*Mean difference*	*p*	*Mean difference*	*p*	*Mean difference*	*p*
CFAI Overall	-0.064	< 0.001	-0.045	0.028	-0.004	1.000	0.019	1.000	0.059	< 0.001	0.041	0.031
CFAI - Communication	-0.080	0.001	-0.006	1.000	0.035	0.379	0.074	0.002	0.115	< 0.001	0.040	0.189
CFAI -Mutuality	-0.046	0.076	-0.084	< 0.001	-0.049	0.023	-0.038	0.243	-0.003	1.000	0.035	0.242
CFAI - Concern	-0.126	< 0.001	-0.017	1.000	0.025	1.000	0.108	< 0.001	0.150	< 0.001	0.042	0.148
CFAI - Conflict	0.007	1.000	0.020	1.000	0.211	< 0.001	0.012	1.000	0.204	< 0.001	0.191	< 0.001
CFAI - Control	-0.070	0.013	-0.141	< 0.001	-0.237	< 0.001	-0.072	0.010	-0.167	< 0.001	-0.095	< 0.001

### Logistic regression analyses

A series of adjusted logistic regression models with age, gender, and education level included as covariates were conducted to examine the effects of the predictors on the likelihood of family functioning specific to each family type. Table 4 represents the odds ratio of each factor associated with family functioning in the five family structures. Never-married individuals with a primary education or lower had significantly lower odds of high family functioning (*aOR* = 0.41, *p* =.002). However, high frequency of family gatherings (*aOR* = 2.24, *p* <.001), high frequency of communication with family members and between generations (*aOR* = 1.39, *p* <.001), and informal social support from family members (*aOR* = 1.58, *p* =.002) were found to be associated with an increase in the odds of experiencing high family functioning for never-married adults. Frequent communication with family members and between generations was the only significant protective factor for elevated likelihood of high family functioning (*aOR* = 1.56, *p* =.006) in married/cohabiting families with no children. Married/cohabiting parents with children who received a primary education or lower demonstrated significantly decreased odds of experiencing high family functioning (*aOR* = 0.71, *p* =.021). In contrast, high frequency of family gatherings (*aOR* = 1.42, *p* =.006) and family communication using modern technologies (*aOR* = 1.12, *p* =.006) increased the odds of high family functioning in two-parent families. For divorced/separated households, being male (*aOR* = 0.65, *p* =.041) had a negative association with family functioning. For widowed households, being male (*aOR* = 0.496, *p* =.003) also had a negative association with family functioning, on the other hand, the presence of informal support from family members (*aOR* = 2.42, *p* <.001) was found to be associated with an increased likelihood of high family functioning.

**Table 4:** Odds ratios of predictors of family functioning by family types

	Family Types									
	Never Married		Married/Cohabiting with No Children		Married/Cohabiting with Children		Divorced/ Separated		Widowed	
	OR (95% CI)	*p*	OR (95% CI)	*p*	OR (95% CI)	*p*	OR (95% CI)	*p*	OR (95% CI)	*p*
**Age (Mean, SD)**	0.999 (0.991, 1.008)	0.080	0.989 (0.970, 1.008)	0.269	0.998 (0.991, 1.005)	0.576	0.994 (0.979, 1.009)	0.433	0.987 (0.971, 1.004)	0.142
**Gender**										
Male	0.835 (0.682, 1.022)	0.861	1.118 (0.722, 1.730)	0.618	0.909 (0.785, 1.052)	0.201	0.654* (0.435, 0.983)	0.041	0.496** (0.313, 0.785)	0.003
Female	1		1		1		1		1	
**Education level**										
Primary education or lower	0.414** (0.234, 0.731)	0.002	0.826 (0.357, 1.911)	0.655	0.709* (0.529, 0.950)	0.021	1.201 (0.375, 3.845)	0.758	0.949 (0.416, 2.165)	0.901
Secondary education	0.909 (0.734, 0.126)	0.382	0.695 (0.417, 1.158)	0.162	0.855 (0.656, 1.115)	0.248	1.263 (0.401, 3.977)	0.690	1.331 (0.578, 3.065)	0.501
Postsecondary education or above	1		1		1		1		1	
**Family meals and gatherings**										
Family meals										
High frequency	0.802 (0.599, 1.073)	0.137	0.849 (0.419, 1.721)	0.649	1.038 (0.816, 1.320)	0.761	0.527 (0.175, 1.592)	0.256	0.831 (0.411, 1.677)	0.604
Low frequency / no gatherings at all	1		1		1		1		1	
Family gatherings										
High frequency	2.242*** (1.685, 2.983)	< 0.001	1.935 (0.964, 3.883)	0.063	1.424** (1.109, 1.828)	0.006	1.457 (0.441, 4.812)	0.537	0.587 (0.272, 1.267)	0.175
Low frequency / no gatherings at all	1		1		1		1		1	
Time spent with parents										
Had spent time with either mother or father	0.777 (0.476, 1.269)	0.313	--		--		--		--	
Did not spend time with parents (including those parents who have died)	1		--		--		--		--	
Time spent with parents or spouse/partner										
Had spent time with either parents or spouse/partner	--		1.522 (0.924, 2.505)	0.099	1.043 (0.840, 1.296)	0.701	1.282 (0.597, 2.756)	0.524	0.377 (0.133, 1.063)	0.065
Did not spend time with either parents or spouse/partner	--		1		1		1		1	
**Family communication**										
Frequency of use of modern technologies to communicate with family members and between generations	1.106 (0.985, 1.242)	0.088	1.018 (0.792, 1.307)	0.890	1.123** (1.034, 1.221)	0.006	1.016 (0.808, 1.276)	0.893	1.062 (0.864, 1.304)	0.568
Frequency of communication with family members and between generations	1.387*** (1.200, 1.603)	< 0.001	1.556** (1.134, 2.134)	0.006	0.998 (0.904, 1.102)	0.966	1.161 (0.937, 1.438)	0.171	1.216 (0.946, 1.563)	0.128
**Social support**										
Informal social support (family members)										
Yes	1.583** (1.178, 2.127)	0.002	0.872 (0.508, 1.496)	0.618	1.054 (0.880, 1.261)	0.569	1.560 (0.895, 2.722)	0.117	2.416*** (1.512, 3.860)	< 0.001
No	1		1		1		1		1	
Informal social support (friends, neighbors, coworkers)										
Yes	0.734 (0.536, 1.006)	0.054	1.663 (0.846, 3.267)	0.140	0.860 (0.671, 1.103)	0.235	0.833 (0.430, 1.612)	0.587	0.999 (0.565, 1.766)	0.997
No	1		1		1		1		1	
Formal social support										
Yes	1.099 (0.726, 1.663)	0.657	1.932 (0.881, 4.239)	0.100	0.846 (0.658, 1.086)	0.189	0.532 (0.273, 1.036)	0.063	0.905 (0.559, 1.465)	0.684
No	1		1		1		1		1	
**Cox & Snell R Square**	0.069		0.090		0.021		0.029		0.078	
**Nagelkerke R Square**	0.093		0.120		0.029		0.040		0.106	
**N**	1702		407		3254		623		565	

*Adjusted for age and education level*

* *p* <.05; ** *p* <.01; *** *p* <.001

*-- denotes analysis not applicable for the family type*

## Discussion

Using four cross-sectional representative household surveys conducted in Hong Kong, the present study is the first to examine changes in family functioning over time. Regarding overall family functioning, although there was no significant difference between the mean scores in 2011 and 2017, there were some fluctuations between 2013 and 2015, implying that there was no obvious trend during the 6-year period. As family functioning is a multidimensional construct consisting of various aspects related to family communication and interaction, this study also measured and looked into the changes in different dimensions of family functioning across time. During the short-term period from 2011 to 2013, there were significant improvements in terms of family communication and concern, followed by decreases in 2015. These declines coincided with the worsening of public mental health after the large-scale social movement (the umbrella movement) in Hong Kong in 2014 [38]. It may be that this deterioration in public mental health spilt over to families, resulting in impaired family functioning, or that the social movement may have had a direct impact on perceptions of family relationships and family functioning, hence causing the declines in family functioning. Although a previous study revealed increased conflicts among families, with disagreements on political views, after the social movement in 2014 [39], the current study found a stable trend of family conflict from 2011 to 2015, suggesting that the overall level of family conflict at the population level was not affected. Apart from fluctuations in some dimensions of family functioning, this study found a steady decline in perceived parental control across the study period, implying an improvement in parenting. However, these explanations of the changes in the population’s family functioning are speculations; additional evidence is needed to verify these hypotheses.

In accordance with our prediction, there were commonalities and differences between predictors of family functioning for different types of family structure. The findings of the current study indicated that frequent communication with family members and between generations is a common predictor of high family functioning shared by most family types (i.e., never married, married/cohabiting with children, and married/cohabiting with no children). However, although family communication is found to be one of the keys to positive family functioning [40], our study revealed that it was not the case for structurally non-intact families due to widowhood, separation, or divorce. Non-intact families may experience higher levels of stress and challenges, which may influence their family communication patterns, and hence do not necessarily benefit from frequent communication with their family members [41]. Interestingly, regarding family communication, married/cohabiting families with children benefited more from frequent communications via technology than from in-person interactions. A possible explanation is that Chinese families generally have close ties with extended family, especially for families with children, frequent communications via technology may enhance intergenerational communications, which in turn enhance family functioning. This finding is also in alignment with a study which shows that the benefits of technology-based communication tend to vary across different family types and different stages of the family life cycle [42]. It is noteworthy that while we found that frequent family communication is associated with higher family functioning in some family types, the study only assessed the frequency, not the quality of family interactions, which is an important variable mediating the association between frequent interaction among family members via technology and higher levels of family functioning [30]. Other factors, such as the technological resources that families have, may also come into play in understanding the association [43]. 

The effects of family gatherings on family functioning depend on family structure. The findings of this study indicated that a high frequency of family gatherings significantly enhanced family functioning for never-married individuals and married/cohabiting families with children. Spending time with parents may be viewed by this group of respondents as more private and intimate, hence improving the perception of family functioning. However, high frequency of family gatherings was associated with lower odds of high family functioning in the widowed group, though the association was non-significant. A possible explanation is that individuals suffering the loss of loved ones may find family gatherings stressful, especially when gatherings take place during holidays and festive seasons. This finding is consistent with the view that family could function as resources as well as situational demands [44]. In contrast to the existing literature suggesting that family meals promote the perception of family communication and functioning [27, 45, 46], the current study did not find a significant association between the frequency of family meals and family functioning for any of the family types. One possible explanation for this finding is that the association may be dependent on factors such as meal type (breakfast, lunch, or dinner) and the quality of interaction during mealtimes, which were not captured in the current study.

Although previous studies consistently found social support to be an important predictor and buffer for mental health [47, 48], the source of social support may play a differential role in different situations. For example, one study found that friend support was associated with psychological well-being for women and family support was associated with self-rated health for men [49]. Another study found that social support from friends, but not from family members, can buffer against suicidal ideation among high-risk women [50]. While previous research shows that intra-familial support has a strong influence on family functioning [31], the current study found that perceived family social support may be more beneficial for individuals who have never married and those who are widowed, but not individuals from other household types. In contrast to previous studies [25, 26], the perception of informal social support from friends and colleagues, as well as formal social support, did not have significant effects on the participants’ perception of family functioning, regardless of the family type. One possible reason could be related to the study’s question items, which only captured whether or not the respondents received support when they had emotional and financial difficulties, but not the quality of the support and other types of support they received.

Finally, being male was associated with lower odds of high family functioning in the two non-intact family types (divorced/separated and widowed). This finding can be interpreted in terms of sex-role expectation. Men who conform to certain masculine norms, such as self-reliance and emotional toughness may be less likely to seek family, friends, or professional support despite the challenges related to the separation and loss [51], resulting in an overall lower level of perceived family functioning.

### Limitations

Several limitations of the current study should be considered when interpreting the results. First, some of the study variables, such as family communication and perception of social support, were single question items, which may result in over- or under-estimation on these variables. Second, the household surveys were cross-sectional and the data of the four surveys could not be linked up at the individual level as they were completed by different respondents at different time points, limiting the study’s ability to delineate the temporal relationship of the study variables. Additionally, the fluctuations in family functioning found between 2013 and 2015 should be interpreted with caution since they could be observed due to differences between participants’ characteristics from the separate samples of household surveys or by chance. Third, only one family member from each household completed the survey; it is possible that different family members within the household would have perceived family functioning differently. Further studies may consider the family as a unit of analysis and collect all family members’ perceptions of family functioning to understand the dynamics of family interactions. Fourth, for the ease of interpretation of the results, we transformed the family functioning variable into high vs. low family functioning. However, such an approach may also lead to a loss of information. Another limitation is that the current study lacks an assessment of individual family members’ outcomes, which prevented us from drawing any conclusion about whether individuals’ outcomes change along with the patterns of family functioning. Also, the original dataset did not have information about the children’s age and sex, which may be confounding factors for family functioning. Furthermore, regarding the generalizability of the findings, since the analyses were completed using Chinese family samples in Hong Kong, future studies may be replicated with other samples which include family members from different age groups in communities with diverse cultural and socio-economic backgrounds for verification.

### Implications

Family functioning is a dynamic construct that may be affected by changes within the family and influences from socio-environmental contexts, such as the COVID-19 pandemic. Future studies with longer-term follow-ups and comprehensive assessments of contextual factors would provide insights to aid understanding of the trends in family functioning. With reference to the Australian experience in collecting routine data of family functioning as one of the indicators of child safety and well-being [13], we encourage future studies to continue to explore how family functioning can be used as an indicator of other social and public health issues, such as family violence and mental health to inform proactive and preventive measures to address these issues. This kind of routine data collection can be easily done by incorporating an appropriate family functioning measurement into existing regular health surveys or health information systems to provide a more holistic view of public health. The study’s findings on the predictors of family functioning suggest that intervention strategies should be tailored for different types of families to address their unique needs. For instance, as family communication is a protective factor for most family types, services aimed at enhancing family communication would be beneficial to the community at large. Enhancing family support is also important, especially for the never married and widowed groups. Our findings also suggest that tailored support for men who experience divorce, separation, and widowhood is needed. Although the current study did not find significant associations of peer support and formal social support with family functioning, this should not be interpreted as indicating that these supportive networks are unimportant. Instead, more research work is needed to enhance our understanding of their roles in family functioning.

## Conclusion

This study provides one of the first pieces of evidence on changes in family functioning at a population level across time. Regarding the protective factors of family functioning, the current study showed that frequent family communication is a protective factor commonly shared by most family types. Future public education and community programs could focus on improving family communication as a way to promote family functioning, which in turn may enhance families’ resilience against a variety of crisis and hardships.

### Electronic supplementary material

Below is the link to the electronic supplementary material.

Supplementary Material 1

## Data Availability

The datasets used and analyzed during the current study are available from the corresponding author on reasonable request.
