# Supplementary material for: Changes in, and factors associated with family functioning: results of four cross-sectional household surveys from 2011 to 2017 in Hong Kong
Source: BMC Public Health. 2024 Jan 11;24:160. doi: 10.1186/s12889-024-17643-6 (PMC10785327; doi:10.1186/s12889-024-17643-6)
Supplement: Supplementary file 1 — Supplementary Material 1 [file 12889_2024_17643_MOESM1_ESM.docx]

**Additional File 1: Changes in family functioning (CFAI) from 2011 to 2017 by family types**

|  | 2011  (n = 2000) | | 2013  (n = 2000) | | 2015  (n = 2000) | | 2017  (n = 2932) | | F-test | p-value |
| --- | --- | --- | --- | --- | --- | --- | --- | --- | --- | --- |
|  | Mean | SD | Mean | SD | Mean | SD | Mean | SD |  |  |
| CFAI Overall |  |  |  |  |  |  |  |  |  |  |
| All families | 3.95 | .53 | 4.02 | .52 | 4.00 | .51 | 3.96 | .45 | 8.370 | <.001 |
| By family types |  |  |  |  |  |  |  |  |  |  |
| 1. Never married | 3.90 | .56 | 3.93 | .53 | 3.89 | .55 | 3.87 | .48 | 1.262 | .286 |
| 1. Married/cohabiting with no children | 3.88 | .49 | 3.98 | .54 | 3.98 | .44 | 3.92 | .47 | 1.388 | .245 |
| 1. Married/cohabiting with children | 4.05 | .48 | 4.11 | .48 | 4.10 | .49 | 4.06 | .40 | 3.818 | .010 |
| 1. Divorced/separated | 3.84 | .59 | 3.93 | .54 | 3.91 | .51 | 3.73 | .49 | 5.742 | 0.001 |
| 1. Widowed | 3.74 | .55 | 3.87 | .59 | 3.86 | .52 | 3.91 | .41 | 3.460 | 0.016 |
| CFAI - Communication |  |  |  |  |  |  |  |  |  |  |
| All families | 4.04 | .62 | 4.12 | .66 | 4.05 | .64 | 4.01 | .62 | 12.782 | <.001 |
| By family types |  |  |  |  |  |  |  |  |  |  |
| 1. Never married | 3.99 | .63 | 4.01 | .69 | 3.92 | .70 | 3.92 | .67 | 2.889 | .034 |
| 1. Married/cohabiting with no children | 4.02 | .53 | 4.14 | .72 | 4.21 | .49 | 4.07 | .56 | 2.618 | .050 |
| 1. Married/cohabiting with children | 4.16 | .55 | 4.23 | .60 | 4.16 | .55 | 4.12 | .53 | 8.015 | <.001 |
| 1. Divorced/separated | 3.87 | .74 | 4.00 | .68 | 3.88 | .70 | 3.69 | .86 | 6.424 | <.001 |
| 1. Widowed | 3.73 | .75 | 3.90 | .85 | 3.84 | .77 | 3.92 | .64 | 2.187 | 0.088 |
| CFAI - Mutuality |  |  |  |  |  |  |  |  |  |  |
| All families | 3.96 | .62 | 4.01 | .61 | 4.04 | .58 | 4.01 | .52 | 6.929 | <.001 |
| By family types |  |  |  |  |  |  |  |  |  |  |
| 1. Never married | 3.92 | .63 | 3.92 | .62 | 3.95 | .59 | 3.94 | .51 | .421 | .738 |
| 1. Married/cohabiting with no children | 3.98 | .56 | 4.02 | .59 | 4.03 | .56 | 4.09 | .56 | 1.023 | .382 |
| 1. Married/cohabiting with children | 4.06 | .60 | 4.09 | .59 | 4.13 | .58 | 4.09 | .49 | 3.087 | .026 |
| 1. Divorced/separated | 3.85 | .66 | 3.97 | .61 | 4.02 | .53 | 3.57 | .56 | 23.305 | <.001 |
| 1. Widowed | 3.58 | .62 | 3.69 | .61 | 3.80 | .56 | 4.05 | .43 | 31.007 | <.001 |
| CFAI - Concern |  |  |  |  |  |  |  |  |  |  |
| All families | 4.08 | .69 | 4.21 | .67 | 4.10 | .65 | 4.06 | .57 | 23.068 | <.001 |
| By family types |  |  |  |  |  |  |  |  |  |  |
| 1. Never married | 4.04 | .71 | 4.13 | .68 | 3.99 | .69 | 3.94 | .64 | 8.259 | <.001 |
| 1. Married/cohabiting with no children | 3.88 | .71 | 4.05 | .80 | 3.87 | .78 | 3.85 | .69 | 1.840 | .139 |
| 1. Married/cohabiting with children | 4.17 | .67 | 4.30 | .62 | 4.22 | .58 | 4.18 | .49 | 9.548 | <.001 |
| 1. Divorced/separated | 4.00 | .69 | 4.10 | .70 | 3.98 | .66 | 3.93 | .53 | 2.603 | 0.051 |
| 1. Widowed | 3.96 | .69 | 4.14 | .77 | 4.03 | .69 | 3.99 | .50 | 2.058 | 0.104 |
| CFAI - Conflict |  |  |  |  |  |  |  |  |  |  |
| All families | 3.70 | .71 | 3.69 | .74 | 3.68 | .70 | 3.49 | .71 | 51.205 | <.001 |
| By family types |  |  |  |  |  |  |  |  |  |  |
| 1. Never married | 3.60 | .74 | 3.56 | .76 | 3.54 | .71 | 3.36 | .72 | 15.154 | <.001 |
| 1. Married/cohabiting with no children | 3.62 | .58 | 3.67 | .80 | 3.64 | .65 | 3.56 | .62 | .812 | .488 |
| 1. Married/cohabiting with children | 3.85 | .64 | 3.83 | .68 | 3.83 | .65 | 3.67 | .64 | 19.010 | <.001 |
| 1. Divorced/separated | 3.48 | .79 | 3.49 | .76 | 3.45 | .74 | 3.16 | .89 | 7.797 | <.001 |
| 1. Widowed | 3.49 | .85 | 3.54 | .83 | 3.52 | .73 | 3.19 | .70 | 10.964 | <.001 |
| CFAI - Control |  |  |  |  |  |  |  |  |  |  |
| All families | 3.99 | .73 | 4.06 | .72 | 4.13 | .77 | 4.23 | .65 | 48.036 | <.001 |
| By family types |  |  |  |  |  |  |  |  |  |  |
| 1. Never married | 3.96 | .74 | 4.00 | .75 | 4.03 | .78 | 4.20 | .68 | 14.415 | <.001 |
| 1. Married/cohabiting with no children | 3.86 | .75 | 4.04 | .76 | 4.17 | .83 | 4.03 | .79 | 3.579 | .014 |
| 1. Married/cohabiting with children | 4.03 | .70 | 4.09 | .70 | 4.16 | .78 | 4.22 | .63 | 15.897 | <.001 |
| 1. Divorced/separated | 4.00 | .77 | 4.06 | .72 | 4.19 | .67 | 4.30 | .61 | 7.614 | <.001 |
| 1. Widowed | 3.96 | .75 | 4.06 | .77 | 4.12 | .75 | 4.37 | .61 | 14.427 | <.001 |
